# Codon Usage Preference and Evolutionary Analysis of Pseudorabies Virus

**DOI:** 10.3390/genes16101155

**Published:** 2025-09-29

**Authors:** Aolong Xiong, Kai Li, Xiaodong Liu, Yunxin Ren, Fuchao Zhang, Xiaoqi Li, Ziqing Yuan, Junhong Bie, Jinxiang Li, Changzhan Xie

**Affiliations:** 1 College of Veterinary Medicine, Chengyang Campus, Qingdao Agricultural University, Qingdao 266109, China; 18254597006@163.com (A.X.); xdliu@qau.edu.cn (X.L.); 18139110469@163.com (X.L.); gillianyzq@163.com (Z.Y.); bjh111@126.com (J.B.); 2 Institute of Urban Agriculture, Chinese Academy of Agricultural Sciences, Chengdu National Agricultural Science & Technology Center, Chengdu 610213, China; likai01@caas.cn; 3 College of Animal & Veterinary Sciences, Southwest Minzu University, Chengdu 610041, China; 17614855357@163.com (Y.R.); zhangfuchao1208@163.com (F.Z.)

**Keywords:** Pseudorabies virus, evolutionary analysis, Bayesian method

## Abstract

**Background:** Pseudorabies virus (PRV), a critical porcine herpesvirus, induces severe diseases in both livestock and wildlife, imposing an incalculable burden and economic losses in livestock production. In this study, we investigated the evolutionary mechanisms and host adaptation strategies of the PRV *gB* gene through genomic alignment. The *gB* gene is highly conserved in PRV, and its encoded gB protein exhibits functional interchangeability across different herpesvirus species. Notably, the *gB* protein elicits the production of both complement-dependent and complement-independent neutralizing antibodies in animals, while also being closely associated with syncytium formation. **Methods:** Phylogenetic analysis and codon usage pattern analysis were performed in this study. A total of 110 *gB* gene sequences were analyzed, which were collected from [2011 to 2024] across the following regions: [Fujian, Shanxi, Guangxi, Guangdong, Chongqing, Henan, Shaanxi, Heilongjiang, Sichuan, Jiangsu, Jilin, Huzhou, Shandong, Hubei, Jiangxi, Beijing, Shanghai, Chengdu (China)], [Budapest, Szeged (Hungary)], [Tokyo (Japan)], [London (United Kingdom)], [Athens (Greece)], [Berlin (Germany)], and [New Jersey (United States)]. **Results:** The *gB* gene of PRV employs an evolutionary “selective optimization” strategy to maintain a dynamic balance between ensuring functional expression and evading host immune pressure, with this core trend strongly supported by its codon usage bias and mutation characteristics. First, the gene exhibits significant codon usage bias [Effective Number of Codons (ENC) = 27.94 ± 0.1528], driven primarily by natural selection rather than mere mutational pressure. Second, phylogenetic analysis shows that the second codon position of *gB* has the highest mutation rate (1.0586)—a feature closely linked to its antigenic variation and immune escape capabilities, further reflecting adaptive evolution against host immune pressure. Additionally, ENC-GC3 plot analysis reveals the complex regulatory mechanisms underlying codon bias formation, providing molecular evidence for the “selective optimization” strategy and clarifying PRV’s core evolutionary path to balance functional needs and immune pressure over time. **Conclusions:** Our study findings deepen our understanding of the evolutionary mechanisms of PRV and provide theoretical support for designing vaccines and assessing the risk of cross-species transmission.

## 1. Introduction

Pseudorabies virus (PRV) is a porcine herpesvirus that belongs to the Alphaherpesvirinae subfamily. Pseudorabies was first detected in cattle in the United States and was identified by Aujeszky in 1902; consequently, the disease is also known as Aujeszky’s disease, and PRV is its etiological agent [1]. PRV primarily induces clinical symptoms such as fever, pruritus, and encephalomyelitis in both livestock and wildlife. While pigs serve as the natural host of PRV, the virus can infect a broad range of mammals, including humans, cats, dogs, goats, and cattle. Notably, all infected hosts other than pigs ultimately succumb to the infection.

PRV has inflicted significant economic damage on the global and Chinese swine industry [2,3]. PRV is classified into two major genotypes: genotype I [4] and genotype II [5]. Notably, PRV exhibits only one serotype; however, virulence varies significantly among different strains, and this variation is regulated by multiple viral genes. Among PRV genes, the glycoprotein B (*gB*) gene—a highly conserved component of the PRV genome—plays a crucial role in viral infection processes [6]. Over time, PRV has co-evolved with its hosts, developing sophisticated strategies to evade host immune surveillance [7].

Prior to this, researchers had studied the codon usage bias between the *US1* gene of PRV and the US1-like genes of 20 reference alphaherpesviruses.

We selected the *gB* gene of PRV for codon analysis owing to its essential role in viral entry, high conservation across strains, and potent immunogenicity [8]. Compared with variable genes (e.g., *gC/gE*), this gene has a stable sequence, making it particularly valuable for evolutionary studies, vaccine development, and cross-genotype comparisons [9].

Codons comprise three nucleotide bases in DNA or RNA and encode specific amino acids.

Codon usage bias refers to the non-random selection and use of specific synonymous codons when encoding the same amino acid [10,11]. This bias is characterized by a significantly higher frequency of certain “optimal codons” relative to other synonymous codons, and it is shaped by multiple factors [12,13].

Host-specific codon usage patterns influence viral gene expression, reflecting the interplay between mutation, selection, and genome structure. Specifically, codon usage affects protein function, translation efficiency, viral replication fitness, and virulence [14]. Previous studies have identified nucleotide homology between the PRV *gB* gene and genes encoding other viral proteins [15], which suggests shared evolutionary origins. In the present study, we leveraged an updated global dataset from the National Center for Biotechnology Information (NCBI) to conduct a comprehensive analysis of the evolution, genetic diversity, and codon usage patterns of the PRV *gB* gene. Our findings advance the understanding of PRV’s molecular evolution, pathogenesis, and functional mechanisms, while also providing valuable insights for broader herpesvirus research.

## 2. Materials and Methods

### 2.1. Genomic Analysis

A literature review was used to compare the nucleotide and amino acid sequences of the *gB* gene [16]. The neighbor-joining method was used to construct phylogenetic trees based on nucleotide sequences. Before performing visual analysis using the iTOL v6 online tool, the sequences were compared using MEGA software (MEGA 5). Table 1 presents the reference sequences.

### 2.2. Phylogeographic Model Analysis

jModelTest was used to select the best-fit evolutionary model (GTR + I + G). A relaxed molecular clock (log-normal) and Bayesian skyline model were applied in BEAST v1.10.4 to estimate the time of the most recent common ancestor and evolutionary rates [17]. Before estimating the adequate population size, BEAST v1.10.4 was used to perform phylogenetic analysis under the GTR + I + G substitution model, a relaxed molecular clock (log-normal distribution), and a coalescent Bayesian skyline. The Markov chain Monte Carlo (MCMC) simulation ran for 100 million generations, sampling every 10,000 steps and parameter logging every 1 million iterations. Tracer v1.7.2 was used to assess convergence, and TreeAnnotator (v1.10.4) was used to deduce the maximum clade credibility.

### 2.3. Codon Usage Indices

#### 2.3.1. Nucleotide Analysis

The nucleotide composition (A, T, G, C) was analyzed, and the GC and AT contents were computed. In addition, the third nucleotide frequencies (A3, C3, T3, G3) and GC content at each codon position (GC1s, GC2s, GC3s) were determined using reference methods [18]. Stop codons (TAA, TAG, TGA) and non-degenerate codons (ATG, TGG) were excluded.

The frequency of the third nucleotide (A3s, C3s, T3s, and G3s) was counted using Codon W (version 1.4.2) (http://codonw.sourceforge.net/ (accessed on 11 June 2025)). GC content (%G + C) refers to the GC content at the first (GC1s), second (GC2s), and third (GC3s) codon positions (http://emboss.toulouse.inra.fr/cgi-bin/emboss/cusp (accessed on 11 June 2025)). The average frequency of GC1s and GC2s (GC12s) is calculatedthrough the use of CAIcal.

#### 2.3.2. Synonymous Codon Bias Analysis Based on Effective Number of Codons (ENC)

ENC is used to quantify synonymous codon usage bias by measuring deviations from random expectations. Unlike other metrics, gene length and amino acid composition do not affect ENC [16]. Therefore, it provides a standardized assessment of codon selection preferences in coding sequences [19].

#### 2.3.3. Analysis of Synonymous Codon Usage Bias Using Relative Synonymous Codon Usage (RSCU)

RSCU was used to analyze synonymous codon usage bias. This approach helps characterize codon selection bias by statistically comparing experimentally observed frequencies against hypothetical equal usage patterns across synonymous codons [20,21]. In this study, RSCU values of highly expressed genes were used to establish reference tables (http://www.bioinformatics.nl/cgi-bin/emboss/cusp (accessed on 13 June 2025)) [16].

#### 2.3.4. Genome-Wide Codon Adaptation Analysis Using Codon Adaptation Index (CAI)

CAI is a powerful indicator to quantify the effect of natural selection on codon usage patterns [22]. CAI helps quantify the effect of natural selection on codon usage [23], ranging from 0 (weak adaptation) to 1 (strong adaptation) [16,24].

#### 2.3.5. Frequency of Optimal Codons (FOP) Analysis

FOP helps identify optimal codons based on tRNA abundance, with higher values indicating stronger adaptation [16,20].

#### 2.3.6. Genome-Wide Codon Usage Profiling via ENC-GC3 Scatter Plot Analysis

ENC-GC3 analysis helps distinguish between mutation-driven and selection-driven biases in codon usage. This helps assess whether there are significant differences in gene distribution under selection pressure compared with the expected distribution [16,25].

## 3. Results

### 3.1. Phylogenetic Analysis of the gB Gene

**Figure 1:**
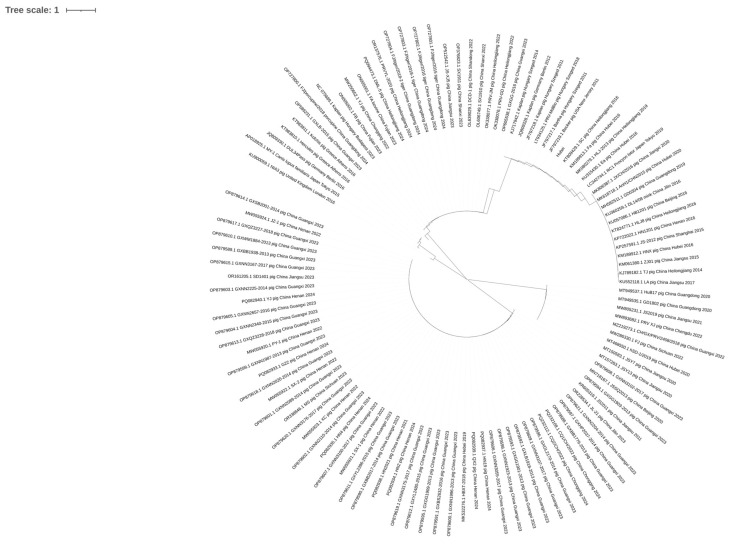
A phylogenetic tree based on all PRV *gB* protein nucleotide sequences.

We constructed a neighbor-joining phylogenetic tree using the nucleotide sequences of the *gB* gene. The analysis was supported by 1000 bootstrap replicates (Figure 1).

### 3.2. Bayesian MCMC-Based Evolutionary Tree

**Figure 2:**
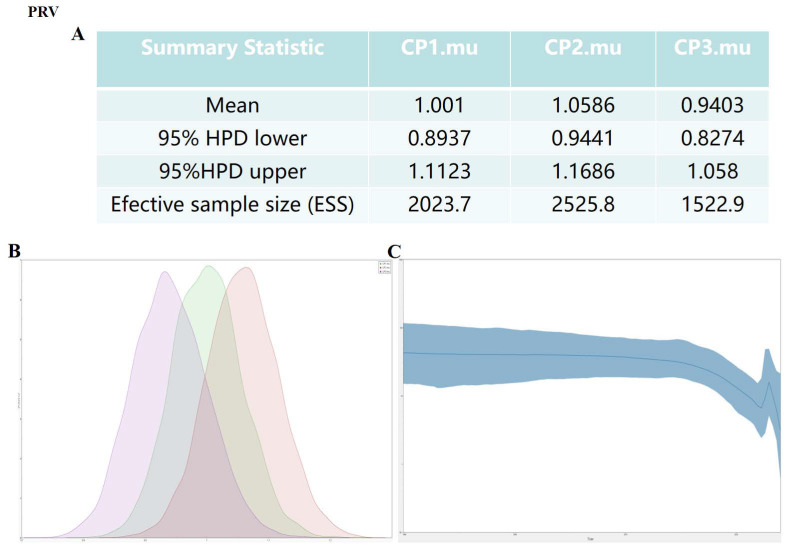
PRV *gB* Codon mutation rate of structural proteins and type I skyline map (**A**,**B**). The codon, mutation rate of PRV structural protein gene was estimated by Bayesian Markov chain method. The codon mutation rate is the result of BEAST running using Trace analysis. (**C**) The dynamic study of the genetic diversity of PRV structural protein genes by IBayesian skyline diagram. The thick, solid line is the median estimate, and the dashed line repressents the 45% confidence interval. The abscissa is time, and the ordinate is the effective population size.

Bayesian MCMC analysis revealed distinct mutation patterns across codon positions in the *gB* gene. The second position exhibited the highest substitution rate (1.0586) (Figure 2A). Despite its frequent synonymous nature, this increased mutation rate at the second codon position suggests potential roles in modulating the mRNA secondary structure or translation kinetics without changing the amino acid sequence. Synonymous codon mutations may also affect mRNA turnover rates, which may be related to their effects on translation kinetics, and may also affect protein-RNA interactions. The temporal reconstruction of PRV population dynamics revealed a significant decrease in effective population size between 2010 and 2020. This potentially reflects the widespread implementation of vaccination programs. However, the subsequent resurgence observed in 2023 suggests the emergence of novel viral variants that can evade existing immune pressures. This underscores the dynamic nature of PRV evolution in response to anthropogenic selection forces (Figure 2B,C).

### 3.3. RSCU Analysis

**Figure 3:**
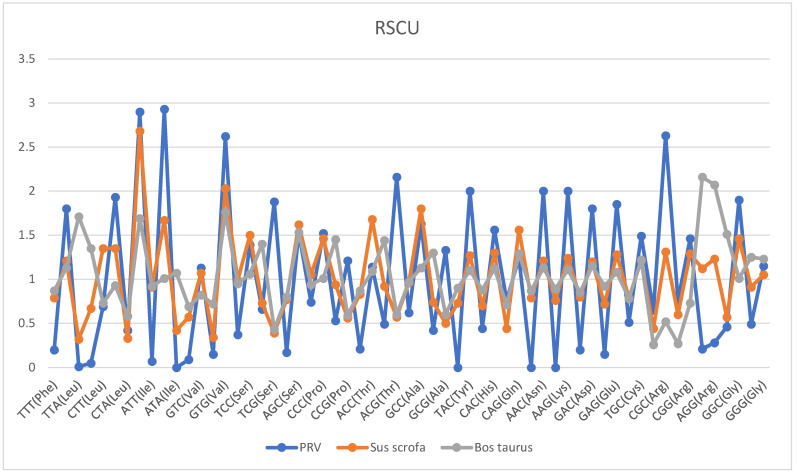
Statistical graph of relative usage rate of synonymous codons.

Table 2 and Figure 3 present the statistical graphs of the relative usage rate of synonymous codons.

In codon usage, PRV exhibits pronounced extreme preferences. For instance, it shows complete avoidance of the three common codons TAT, AAT, and AAA, relying exclusively on TAC, AAC, and AAG. Meanwhile, it demonstrates exceptionally high usage frequency for Thr (ACG) and Ser (TCG), while showing minimal usage frequency for Thr (ACT) and Ser (AGT). In contrast, as a higher-order eukaryote, Sus scrofa demonstrates more balanced codon usage. Not only does it lack any codons with zero frequency, but the usage frequencies among synonymous codons corresponding to these amino acids also maintain relative equilibrium.

Bos taurus as a non-natural host for PRV—they cannot harbor the virus for a long term after infection and eventually die. There exists a significant codon preference mismatch between cattle and PRV, which is fully reflected from the levels of core codon selection to base-ending preference: In terms of core codon usage, the key codon choices of the two are completely opposite. For instance, PRV relies heavily on CTC/CTG for leucine (Leu) (Relative Synonymous Codon Usage, RSCU = 1.93/2.9), ATC for isoleucine (Ile) (RSCU = 2.93), and CGC for arginine (Arg) (RSCU = 2.63). In contrast, cattle prefer Leu-TTA/TTG (RSCU = 1.71/1.35)—codons that PRV avoids, use Ile-ATA (RSCU = 1.07)—a codon that PRV does not use at all, and rely highly on Arg-AGA/AGG (RSCU = 2.16/2.07)—codons that PRV strongly evades. In terms of base-ending preference, cattle have a much higher acceptance of A/T-ending codons than PRV. For example, cattle prefer ACA (A-ending, RSCU = 1.44) for threonine (Thr) and GCA (A-ending, RSCU = 1.3) for alanine (Ala). However, PRV strongly avoids A/T-ending codons and only uses Thr-ACG (G-ending, RSCU = 2.16) and Ala-GCC (C-ending, RSCU = 1.63). This comprehensive mismatch leads to two critical consequences: First, PRV struggles to bind to the host tRNA pool in cattle, significantly reducing the translation efficiency of viral proteins. Second, the mismatched viral mRNAs are more likely to be recognized as foreign substances by the cattle’s immune system, triggering a strong immune response. Ultimately, these effects result in the rapid death of cattle after PRV infection, making them unable to act as effective transmission hosts for PRV.

To evade RNA instability caused by AU-rich sequences, PRV reduces AU content at the codon level to minimize AU-rich regions in its RNA from the source. Specifically, PRV rarely uses AT-rich codons (containing two A/T bases), such as TTT (for Phe), TTA (for Leu), ATT/ATA (for Ile), GTT/GTA (for Val), TAT (for Tyr), AAT (for Asn), AAA (for Lys), GAT (for Asp), and GAA (for Glu), with their usage frequencies mostly 0 or ≤0.21. Instead, it strongly prefers synonymous codons with higher GC content, like TTC (Phe), CTC/CTG (Leu), ATC (Ile), and GTC/GTG (Val), whose frequencies are mostly ≥1.13. In contrast, the host (Sus scrofa) maintains a certain usage of these AT-rich codons (e.g., TTT: 0.79, ATT: 0.91, TAT: 0.73). This comparison clearly shows PRV avoids RNA instability by abandoning AT-rich codons.

RSCU is an essential indicator for measuring codon usage bias. An RSCU value of >1 indicates positive codon bias, an RSCU value of <1 indicates negative bias, and an RSCU values of 1 suggest random usage [26]. According to the curve in the figure, the RSCU value of CTA, ATT, CGC and GGC is >1, indicating that the codon is preferred

### 3.4. Nucleotide Bias in PRV Genotypes

Cytosine (C) was identified as the most abundant nucleotide (37.4% ± 0.19%), with C3s exhibiting the highest frequency among synonymous codons (0.67 ± 0.0027) (Table 3).

### 3.5. Codon Bias Measurement

The ENC value of the *gB* gene was 27.94 ± 0.1528. This indicates strong codon bias (range: 20–61; lower values denote more substantial bias) [27]. Overall, based on the numerical point of view presented in Table 3, the *gB* gene exhibits significant codon usage bias.

### 3.6. Synonymous and Optimal Codon Analysis

**Figure 4:**
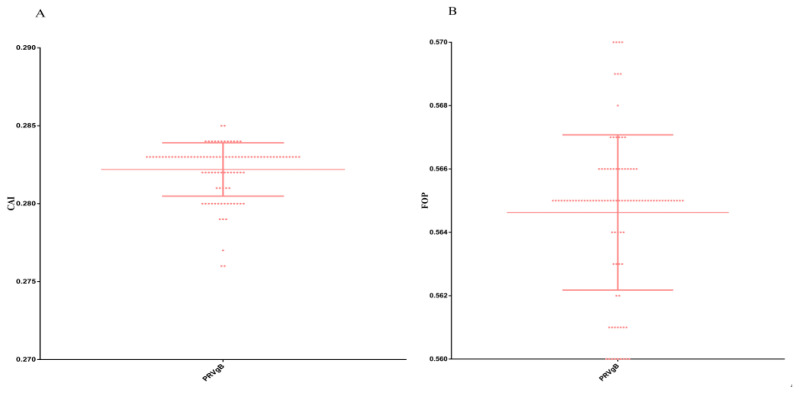
(**A**): Scatter plot of PRV *gB* structural protein gene CAI. (**B**): Scatter plot of PRV *gB* structural protein gene FOP. The dots in the figure show the distribution of different genes.

Figure 4A,B illustrate the scatter plots of the structural protein gene of PRV *gB* via CAI and FOP, respectively. The higher the CAI, the higher the expression level of the exogenous gene within the host. The scatter plot shows that the CAI value is 0.28 and the FOP value is 0.56. This indicates that the gene’s codon usage pattern does not match the host’s preferences well. The FOP value of 0.56 suggests that while the gene’s codon usage tends to favor the host’s optimal codons, the degree of optimization is moderate.

### 3.7. ENC-GC3 Plot Analysis

**Figure 5:**
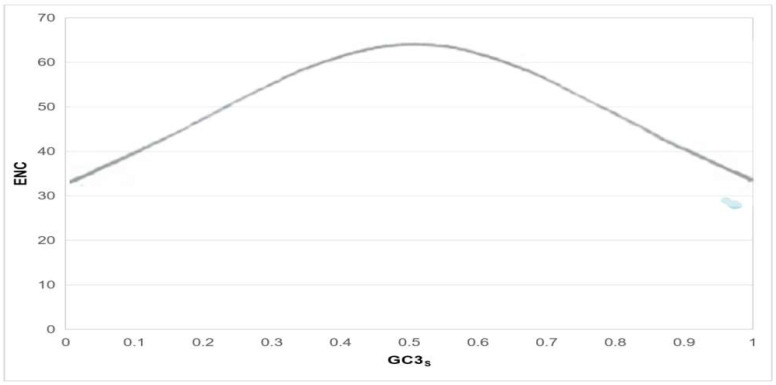
PRV *gB* ENC The relationship between GC3.In this figure, each blue dot represents a single gene, and its position in the graph is jointly determined by the horizontal axis (GC3s) and the vertical axis (ENC). The horizontal axis, GC3s, reflects the G + C content at the third position of the gene’s codons, which can indicate the base composition preference at the third position of synonymous codons. The vertical axis, ENC (Effective Number of Codons), is used to measure the degree of codon usage bias of the gene; a lower ENC value indicates a stronger codon usage bias of the gene and a more concentrated range of codons it relies on. Thus, the specific position of each blue dot can comprehensively represent the core characteristics of the corresponding gene in terms of both base composition preference and codon usage bias.

As illustrated in Figure 5, the genes plotted below the expected curve exhibited significant natural selection pressure on codon usage.

## 4. Discussion and Conclusions

These findings not only deepen our understanding of the evolutionary mechanisms of PRV but also provide essential theoretical support for designing vaccines based on codon optimization.

Sharp et al. [28] have hypothesized that viral genes typically use codons that match their host’s tRNA repertoire to optimize translation efficiency. Similarly, Chen et al. [22] analyzed porcine epidemic diarrhea virus and reported that natural selection is the primary driving force behind codon preference. These findings are consistent with those of previous studies on codon usage patterns in herpesviruses. Overall, these studies support the conclusion of our research that PRV adapts to the host cell environment via codon optimization, thereby enhancing its replication and transmission capabilities.

In this study, we systematically analyzed the codon usage patterns of the *gB* gene in PRV. We noted that unique codon preference characteristics were formed during virus evolution and their biological significance. Codon optimization fragments have been validated by replacing the PRV gene with the US3 gene [29]. Translational optimization may drive the preferential selection of certain synonymous codons, reflecting adaptation to the specific biological constraints of the organism [30].

Previous studies have shown that mRNA stability is closely linked to codon type: stable mRNAs are rich in optimal codons, while unstable ones are dominated by non-optimal codons. For viruses, this translates to a specific adaptive strategy: they reduce AU content at the codon level to minimize the formation of AU-rich sequences in their RNA from the source. Essentially, viruses avoid non-optimal AT-rich codons and prefer optimal codons with higher GC content, thereby evading the mRNA degradation risk caused by AU-rich sequences. This regulatory mechanism ensures the stability of viral RNA, laying the foundation for successful viral protein translation and progeny replication [31].

Mazumdar P et al. [32] compared grass and non-grass monocots to elucidate their distinct evolutionary patterns. Codon optimization technology has been widely applied in viral vaccine development; for instance, Xu et al. [33] performed codon optimization on the PRV US3 gene, successfully constructing a live attenuated vaccine candidate strain. This strain significantly reduced viral virulence while preserving immunogenicity. Similarly, Ma et al. [34] proposed that codon usage preferences in the PRV genome may influence the virus’s virulence and host range. These studies demonstrate that by targeting codon optimization or de-optimization, researchers can design safer and more effective PRV vaccines. The codon usage preferences of the PRV *gB* gene identified in the present study thus provide important references for future vaccine design.

While studying the evolution of other viruses to understand the trend of codon usage during the novel coronavirus epidemic, some researchers conducted related codon analysis [35]. They noted that the codon preferences of the PRV *gB* gene significantly differ from those of other herpesviruses (such as HSV-1) and RNA viruses [such as porcine epidemic diarrhea virus and classical swine fever virus (CSFV)]. This difference suggests that viruses have different adaptive strategies to host translation mechanisms. Furthermore, the CAI value of the PRV *gB* gene (~0.28) is lower than that of some highly expressed host genes; this suggests that the virus avoids being over-recognized by the host immune system by moderately deviating from the host’s codon usage patterns.

According to data, the second codon site of porcine circovirus (PRV) *gB* gene may play a significant role in promoting antigenic drift and evading immune systems. For example, Butt et al. [26] discovered that the Zika virus adapts to different hosts and vectors via alterations in codon usage preferences. The high mutation rate of the PRV *gB* gene may facilitate its sustained evolution under vaccine pressure, offering a theoretical foundation for future vaccine updates and adjustments in control strategies.

While the present study revealed the codon preferences of the PRV *gB* gene and their evolutionary significance, several questions remain unexplored. For example, is there conservation in the codon usage patterns of PRV compared with other herpesviruses (such as HSV or varicella–zoster virus)? Additional experimental validation of the expression efficiency and immune reactivity of codon-optimized *gB* protein can provide more definitive evidence for vaccine development.

The evolutionary pathway of the complete genomic DNA sequence of PRV has not been determined. Using codon optimization techniques after understanding the codon preferences of viruses can help produce innovative PRV attenuated live vaccine candidates. Our study findings advance our understanding of the evolution of PRV and facilitate vaccine design and cross-species transmission risk assessment. Finally, codon deoptimization may serve as a strategy to develop attenuated PRV vaccines.

## Figures and Tables

**Table 1:** Reference sequence information.

Sequence Name	GenBank Accession NO.	Year	Area
CQ2/CH/2022	PQ232112.1	2024	China
CQ1/CH/2022	PQ232108.1	2024	China
DML-5	PQ094473.1	2024	China
YJ	PQ082940.1	2024	China
QX2	PQ082939.1	2024	China
HN2021	PQ082938.1	2021	China
HN19	PQ082937.1	2024	China
HN4	PQ082935.1	2024	China
HN2	PQ082934.1	2024	China
G22	PQ082933.1	2024	China
MS	OR338846.1	2023	China
JL-21	OR228534.1	2023	China
SD1401	OR161205.1	2023	China
PRVYL-2020	OR137576.1	2024	China
GXNN2204-2014	OP879621.1	2023	China
GXNN3176-2017	OP879620.1	2023	China
GXNN3175-2017	OP879619.1	2023	China
GXNN2020-2014	OP879618.1	2023	China
GXQZ3227-2018	OP879617.1	2023	China
GXNN3167-2017	OP879615.1	2023	China
GXSB2001-2014	OP879614.1	2023	China
GXQZ3228-2018	OP879613.1	2023	China
GXYL2480-2015	OP879612.1	2023	China
GXYL2396-2015	OP879611.1	2023	China
GXWM1884-2013	OP879610.1	2023	China
GXNN3107-2017	OP879609.1	2023	China
GXNN3102-2017	OP879608.1	2023	China
GXNN3100-2017	OP879607.1	2023	China
GXNN3055-2017	OP879606.1	2023	China
GXNN2657-2016	OP879605.1	2023	China
GXNN2340-2015	OP879604.1	2023	China
GXNN2225-2014	OP879603.1	2023	China
GXNN2110-2014	OP879602.1	2023	China
GXNN2089-2014	OP879601.1	2023	China
GXNN1996-2013	OP879600.1	2023	China
GXNN1987-2013	OP879599.1	2023	China
GXNN1923-2013	OP879598.1	2023	China
GXHP2037-2014	OP879597.1	2023	China
GXGL2170-2014	OP879596.1	2023	China
GXGG1909-2013	OP879595.1	2023	China
GXGG1903-2013	OP879594.1	2023	China
GXGG1901-2013	OP879593.1	2023	China
GXLB1918-2013	OP879592.1	2023	China
GXBS2632-2016	OP879591.1	2023	China
GXBB2017-2014	OP879590.1	2023	China
GXBB1938-2013	OP879589.1	2023	China
GXBB1776-2013	OP879588.1	2023	China
FJ/tiger/2018-2	OP727804.1	2024	China
FJ/tiger/2018-1	OP727803.1	2024	China
FJ/tiger/2016	OP727802.1	2024	China
FJ/tiger/2015	OP727801.1	2024	China
FJ/porcupine/2018	OP727800.1	2024	China
GXGG-2016	OP605538.1	2023	China
GXLB-2015	OP589231.1	2023	China
JS-XJ5	OP512542.1	2023	China
SX1911	OP376823.1	2023	China
FB	ON005002.1	2023	China
FA	ON005001.1	2023	China
DCD-1	OL639029.1	2022	China
SX1910	OL606749.1	2022	China
PRV-JM	OK338077.1	2022	China
PRV-GD	OK338076.1	2022	China
Kaplan	NC_075689.1	2023	Hungary
CH/GX/PRV/2408/2018	MZ219273.1	2022	China
XJ	MW893682.1	2022	China
_JS2019	MW805231.1	2021	China
FJ	MW286330.1	2022	China
YJ	MW250652.1	2022	China
JZ-1	MW055924.1	2022	China
XC	MW055923.1	2022	China
SX-2	MW055922.1	2022	China
SX-1	MW055921.1	2022	China
PY-1	MW055920.1	2022	China
HuB17	MT949537.1	2020	China
GD1802	MT949535.1	2020	China
hSD-1/2019	MT468550.1	2020	China
JSY13	MT157263.1	2020	China
JSY7	MT150583.1	2020	China
JSSQ2013	MN718167.1	2020	China
JX/CH/2016	MK806387.1	2020	China
AnH1/CHN2015	MK618718.1	2020	China
HBXT-2018	MK532276.1	2019	China
HLJ-2013	MK080279.1	2019	China
GD0304	MH582511.1	2019	China
PRV-MdBio	LT934125.1	2018	Hungary
RC1	LC342744.1	2019	Japan
Ea(Hubei)	KX423960.1	2017	China
NIA3	KU900059.1	2016	United Kingdom
LA	KU552118.1	2017	China
DL14/08	KU360259.1	2016	China
Ea	KU315430.1	2016	China
HB1201	KU057086.1	2016	China
Kolchis	KT983811.1	2016	Greece
Hercules	KT983810.1	2016	Greece
HLJ8	KT824771.1	2016	China
SC	KT809429.1	2016	China
JS2011	KR605319.1	2011	China
HN1201	KP722022.1	2016	China
JS-2012	KP257591.1	2015	China
Fa	KM189913.1	2016	China
HNX	KM189912.1	2016	China
ZJ01	KM061380.1	2015	China
TJ	KJ789182.1	2014	China
Kaplan	KJ717942.1	2014	Hungary
DUL34Pass	JQ809330.1	2016	Germany
Kaplan	JQ809328.1	2012	Germany
Becker	JF797219.1	2011	USA
Kaplan	JF797218.1	2011	Hungary
Bartha	JF797217.1	2011	Hungary
MY-1	AP018925.1	2015	Japan

**Table 2:** Properties of structural protein genes from PRV strains relative synonymous codon usage analysis in this study (Potential hosts are displayed in bold).

Categories	PRV	Sus Scrofa	Bos Taurus
**TTT(Phe)**	0.2	0.79	0.87
**TTC(Phe)**	1.8	1.21	1.13
**TTA(Leu)**	0.01	0.32	1.71
**TTG(Leu)**	0.05	0.67	1.35
**CTT(Leu)**	0.69	1.35	0.73
**CTC(Leu)**	1.93	1.35	0.93
**CTA(Leu)**	0.42	0.33	0.58
**CTG(Leu)**	2.9	2.68	1.69
**ATT(Ile)**	0.07	0.91	0.92
**ATC(Ile)**	2.93	1.67	1.01
**ATA(Ile)**	0	0.42	1.07
**GTT(Val)**	0.09	0.57	0.69
**GTC(Val)**	1.13	1.07	0.82
**GTA(Val)**	0.15	0.34	0.72
**GTG(Val)**	2.62	2.03	1.76
**TCT(Ser)**	0.37	0.99	0.95
**TCC(Ser)**	1.39	1.5	1.06
**TCA(Ser)**	0.66	0.73	1.4
**TCG(Ser)**	1.88	0.39	0.43
**AGT(Ser)**	0.17	0.77	0.8
**AGC(Ser)**	1.53	1.62	1.53
**CCT(Pro)**	0.74	1.05	0.94
**CCC(Pro)**	1.52	1.46	1.01
**CCA(Pro)**	0.53	0.94	1.45
**CCG(Pro)**	1.21	0.56	0.59
**ACT(Thr)**	0.21	0.83	0.87
**ACC(Thr)**	1.14	1.68	1.09
**ACA(Thr)**	0.49	0.92	1.44
**ACG(Thr)**	2.16	0.57	0.6
**GCT(Ala)**	0.62	0.96	0.97
**GCC(Ala)**	1.63	1.8	1.13
**GCA(Ala)**	0.42	0.74	1.3
**GCG(Ala)**	1.33	0.5	0.6
**TAT(Tyr)**	0	0.73	0.9
**TAC(Tyr)**	2	1.27	1.1
**CAT(His)**	0.44	0.7	0.88
**CAC(His)**	1.56	1.3	1.12
**CAA(Gln)**	0.76	0.44	0.71
**CAG(Gln)**	1.24	1.56	1.29
**AAT(Asn)**	0	0.79	0.87
**AAC(Asn)**	2	1.21	1.13
**AAA(Lys)**	0	0.76	0.89
**AAG(Lys)**	2	1.24	1.11
**GAT(Asp)**	0.2	0.8	0.85
**GAC(Asp)**	1.8	1.2	1.15
**GAA(Glu)**	0.15	0.72	0.92
**GAG(Glu)**	1.85	1.28	1.08
**TGT(Cys)**	0.51	0.79	0.78
**TGC(Cys)**	1.49	1.21	1.22
**CGT(Arg)**	0.65	0.44	0.26
**CGC(Arg)**	2.63	1.31	0.52
**CGA(Arg)**	0.76	0.6	0.27
**CGG(Arg)**	1.46	1.29	0.73
**AGA(Arg)**	0.21	1.12	2.16
**AGG(Arg)**	0.28	1.23	2.07
**GGT(Gly)**	0.46	0.57	1.51
**GGC(Gly)**	1.9	1.46	1.01
**GGA(Gly)**	0.49	0.91	1.25
**GGG(Gly)**	1.15	1.05	1.23

**Table 3:** Properties of structural protein genes from PRV *gB* strains analyzed in this study (mean value ± SD).

Categories	PRV
%A	15.1 ± 0.31
%C	37.4 ± 0.19
%T	13.6 ± 0.08
%G	33.8 ± 0.47
A-3	10.4 ± 7.86
C-3	43.1 ± 11.52
T-3	10 ± 6.5
G-3	36.5 ± 3.07
A3_S_	0.01 ± 0.0013
C3_S_	0.67 ± 0.0027
T3_S_	0.02 ± 0.0011
G3_S_	0.50 ± 0.0022
%G + C	0.71± 0.0005
GC3_S_	0.97 ± 0.0016
ENC	27.94 ± 0.1528

## Data Availability

The original contributions presented in this study are included in the article. Further inquiries can be directed to the corresponding author(s).

## References

[1] Pomeranz L.E., Reynolds A.E., Hengartner C.J. (2005). Molecular biology of pseudorabies virus: Impact on neurovirology and veterinary medicine. Microbiol. Mol. Biol. Rev..

[2] Ai J.W., Weng S.S., Cheng Q., Cui P., Li Y.J., Wu H.L., Zhu Y.M., Xu B., Zhang W.H. (2018). Human Endophthalmitis Caused By Pseudorabies Virus Infection, China, 2017. Emerg. Infect. Dis..

[3] Liu Q., Wang X., Xie C., Ding S., Yang H., Guo S., Li J., Qin L., Ban F., Wang D. (2022). Erratum to: A Novel Human Acute Encephalitis Caused by Pseudorabies Virus Variant Strain. Clin. Infect. Dis..

[4] Yu Z., Tong W., Zheng H., Li L., Li G., Gao F., Wang T., Liang C., Ye C., Wu J. (2017). Variations in glycoprotein B contribute to immunogenic difference betw een PRV variant JS-2012 and Bartha-K61. Vet. Microbiol..

[5] Yao J., Li J., Gao L., He Y., Xie J., Zhu P., Zhang Y., Zhang X., Duan L., Yang S. (2022). Epidemiological Investigation and Genetic Analysis of Pseudorabies Vir us in Yunnan Province of China from 2017 to 2021. Viruses.

[6] Avitabile E., Lombardi G., Gianni T., Capri M., Campadelli-Fiume G. (2004). Coexpression of UL20p and gK inhibits cell-cell fusion mediated by herpes simplex virus glycoproteins gD, gH-gL, and wild-type gB or an endocytosis-defective gB mutant and downmodulates their cell surface expression. J. Virol..

[7] Wang C., Li L., Zhai X., Chang H., Liu H. (2024). Evasion of the Antiviral Innate Immunity by PRV. Int. J. Mol. Sci..

[8] Otsuka H., Xuan X., Shibata I., Mori M. (1996). Protective immunity of bovine herpesvirus-1 (BHV-1) recombinants which express pseudorabies virus (PRV) glycoproteins gB, gC, gD and gE. J. Vet. Med. Sci..

[9] Cao Z., Zhang K., Zhang H., Zhang H., Yu Y., Yin D., Shan H., Qin Z. (2022). Efficacy of a gB + gD-based subunit vaccine and the adjuvant granulocy te-macrophage colony stimulating factor for pseudorabies virus in rabb its. Front. Microbiol..

[10] Parvathy S.T., Udayasuriyan V., Bhadana V. (2022). Codon usage bias. Mol. Biol. Rep..

[11] Grantham R., Gautier C., Gouy M., Mercier R., Pavé A. (1980). Codon catalog usage and the genome hypothesis. Nucleic Acids Res..

[12] Wu H., Bao Z., Mou C., Chen Z., Zhao J. (2020). Comprehensive Analysis of Codon Usage on Porcine Astrovirus. Viruses.

[13] Wu X.M., Wu S.F., Ren D.M., Zhu Y.P., He F.C. (2007). The analysis method and progress in the study of codon bias. Yi Chuan.

[14] Sharp P.M., Matassi G. (1994). Codon usage and genome evolution. Curr. Opin. Genet. Dev..

[15] Robbins A.K., Dorney D.J., Wathen M.W., Whealy M.E., Gold C., Watson R.J., Holland L.E., Weed S.D., Levine M., Glorioso J.C. (1987). The pseudorabies virus gII gene is closely related to the gB glycoprotein gene of herpes simplex virus. J. Virol..

[16] Xie C., Tao Y., Zhang Y., Zhang P., Zhu X., Ha Z., Zhang H., Xie Y., Xia X., Jin N. (2022). Codon Usage for Genetic Diversity, and Evolutionary Dynamics of Novel Porcine Parvoviruses 2 through 7 (PPV2-PPV7). Viruses.

[17] Drummond A.J., Rambaut A. (2007). BEAST: Bayesian evolutionary analysis by sampling trees. Bmc Evol. Biol..

[18] PuigBò P., Bravo I.G., Garcia-Vallve S. (2008). CAIcal: A combined set of tools to assess codon usage adaptation. Biol. Direct.

[19] Wright F. (1990). The ‘effective number of codons’ used in a gene. Gene.

[20] Sharp P.M., Tuohy T.M., Mosurski K.R. (1986). Codon usage in yeast: Cluster analysis clearly differentiates highly and lowly expressed genes. Nucleic Acids Res..

[21] Chen Y., Li X., Chi X., Wang S., Ma Y., Chen J. (2017). Comprehensive analysis of the codon usage patterns in the envelope glycoprotein E2 gene of the classical swine fever virus. PLoS ONE.

[22] Chen Y., Chen Y.F. (2014). Analysis of synonymous codon usage patterns in duck hepatitis A virus: A comparison on the roles of mutual pressure and natural selection. Virusdisease.

[23] Chen Y., Shi Y., Deng H., Gu T., Xu J., Ou J., Jiang Z., Jiao Y., Zou T., Wang C. (2014). Characterization of the porcine epidemic diarrhea virus codon usage bias. Infect. Genet. Evol..

[24] Sharp P.M., Li W.H. (1987). The codon Adaptation Index—A measure of directional synonymous codon usage bias, and its potential applications. Nucleic Acids Res..

[25] Majeed A., Kaur H., Bhardwaj P. (2020). Selection constraints determine preference for A/U-ending codons in Taxus contorta. Genome.

[26] Butt A.M., Nasrullah I., Qamar R., Tong Y. (2016). Evolution of codon usage in Zika virus genomes is host and vector specific. Emerg. Microbes Infect..

[27] Vicario S., Moriyama E.N., Powell J.R. (2007). Codon usage in twelve species of Drosophila. Bmc Evol. Biol..

[28] Sharp P.M., Cowe E., Higgins D.G., Shields D.C., Wolfe K.H., Wright F. (1988). Codon usage patterns in Escherichia coli, Bacillus subtilis, Saccharomyces cerevisiae, Schizosaccharomyces pombe, Drosophila melanogaster and Homo sapiens; a review of the considerable within-species diversity. Nucleic Acids Res..

[29] Xu M., Wang Y., Liu Y., Chen S., Zhu L., Tong L., Zheng Y., Osterrieder N., Zhang C., Wang J. (2023). A Novel Strategy of US3 Codon De-Optimization for Construction of an Attenuated Pseudorabies Virus against High Virulent Chinese Pseudorabies Virus Variant. Vaccines.

[30] Arella D., Dilucca M., Giansanti A. (2021). Codon usage bias and environmental adaptation in microbial organisms. Mol. Genet. Genom..

[31] Presnyak V., Alhusaini N., Chen Y., Martin S., Morris N., Kline N., Olson S., Weinberg D., Baker K.E., Graveley B.R. (2015). Codon optimality is a major determinant of mRNA stability. Cell.

[32] Mazumdar P., Binti Othman R., Mebus K., Ramakrishnan N., Ann Harikrishna J. (2017). Codon usage and codon pair patterns in non-grass monocot genomes. Ann. Bot..

[33] Xu M., Zhu L., Ge A., Liu Y., Chen S., Wei Z., Zheng Y., Tong L., Wang Z., Fei R. (2023). Construction of pseudorabies virus variant attenuated vaccine: Codon deoptimization of US3 and UL56 genes based on PRV gE/TK deletion strain. Front. Microbiol..

[34] Ma X.R., Xiao S.B., Fang L.R., Chen H.C. (2005). Bias of base composition and codon usage in pseudorabies virus genes. Yi Chuan Xue Bao.

[35] Wu X., Shan K.J., Zan F., Tang X., Qian Z., Lu J. (2023). Optimization and Deoptimization of Codons in SARS-CoV-2 and Related Implications for Vaccine Development. Adv. Sci..

